# Insights of Healthcare Professionals into Medical Writing Support at a Tertiary Care Hospital in Saudi Arabia

**DOI:** 10.7759/cureus.59190

**Published:** 2024-04-28

**Authors:** Shahad AlOtaiby, Farah AlOtaiby, Adnan AlMaghlouth, Sara AlNassar

**Affiliations:** 1 Research Center, King Fahad Medical City, Central Second Health Cluster, Ministry of Health, Riyadh, SAU; 2 Clinical Pharmacy, King Abdulaziz University, Jeddah, SAU; 3 Research Center, King Faisal Specialist Hospital & Research Centre, Riyadh, SAU

**Keywords:** saudi arabia, health care professionals, questionnaire, ghost-writing, medical writing

## Abstract

Background and aims: The demand for medical writing is on the rise in academic and medical entities worldwide. However, a huge disparity in the perception of professional medical writing arises from inadequate education and regularity in service quality and potential ethical challenges. Hence, we aimed to examine the knowledge, attitude, and perception toward professional medical writing support (PMWS) of healthcare professionals at King Fahad Medical City (KFMC).

Methods: An observational cross-sectional study was conducted using a questionnaire that was validated for its accuracy and clarity by an expert panel in academic integrity. The self-administered questionnaires were distributed to 200 healthcare professionals from a broad range of specialties throughout KFMC. The socio-demographic characteristics and healthcare professionals’ knowledge, attitudes, and perceptions were recorded. The responses were quantitatively evaluated using a 5-point Likert Scale and analyzed using the Statistical Package for Social Sciences 25 (IBM, New York, United States).

Results: 162 healthcare professionals in KFMC filled in the questionnaire completely. Most respondents agreed that it was acceptable for medical writers to help with writing publications and that they offered a valuable service. Concerning association with socio-demographic characteristics, the mean score of knowledge was found to be significantly related to those with age ≥45 years (p<0.001), PhD degree (p=0.044), >5 years of research experience (p<0.001), and being a faculty/consultant (p=0.005). No significant association was found with the mean total score of attitude. Participants having >5 years of research experience were found to have a higher significant level of perception (p=0.004).

Conclusions: Our study highlights that PMWS is relatively well-utilized and perceived positively within KFMC. There is a need for further research and education of ethics regarding the use of PMWS.

## Introduction

Like other branches of science, medical advances have been made possible by a constant stream of new information from ongoing medical research that feeds into growing medical knowledge [1]. Communicating with such a diverse audience requires a universally standardized language, with linguistic and scientific precision, to allow different people worldwide to comprehend the latest advancements. As the most globally used language in medicine and science, the English language has become the lingua franca for the scientific community [2]. However, for non-native English speakers, mastering the art of writing in English can be challenging [3]. This difficulty in writing accurately in English can be problematic as non-native English speakers account for approximately 50% of authors of scientific peer-reviewed publications [4]. In addition, the use of medical terminology can further complicate this process, which sets the term “medical writing” apart from other writing skills [4]. 

Professional medical writing support (PMWS) can aid in ensuring that English writing is grammatically correct and is being hired worldwide by non-native English speakers to improve their text's readability [5]. Within Saudi Arabia, the use of PMWS has increased in the past few years given the expansion of the scientific research culture and the eagerness of researchers to publish their research [6]. Among developing countries, there is an increased demand for publishing due to various reasons including career development, a greater opportunity for international collaboration, and monetary incentives [7]. It is also worth noting that writing allows healthcare practitioners to develop their communication skills and critical thinking ability and is not only an approach to providing information. Moreover, the value that PMWS brings to manuscript development across a broad range of journals may play a catalytic role in raising the quality of clinical trial reporting [7].

However, misconduct related to scientific writing such as “ghostwriting” has been reported and it is defined as the activity of writing books, articles, etc. for another person to publish under their name [5,8,9]. This has been considered to be unethical by several associations such as the American Medical Writers Association (AMWA) and the European Medical Writers Association (EMWA) [10-13]. According to EMWA, ghostwriting is “what happens when someone writes a paper for publication in the medical literature, and neither the identity of the writer nor the funding source of the writing is disclosed to the reader” [14]. A professional medical writer assisting the author must adhere to strict ethical guidelines for medical writing and the original author remains in control of the written piece [15,16].

Owing to stringent regulations and increased awareness of its hazard to public health, ghostwriting has become less prevalent in recent years [17]. Hamilton and Jacobs et. al. identified a continuous decrease in the rate of manuscripts with undisclosed contributions from 62% in 2005 vs 42% in 2008 to 34% in 2014 [10,18]. Despite the encouraging 44% decrease in the ghostwriting rate in nine years since 2005 yet, 34% was still unacceptably high [18].

A study conducted by Badreldin et al. in Saudi Arabia reported insufficient knowledge regarding authorship guidelines and unethical practices involved in scientific publication among health science students and recommended universities and research centers to measures to increase awareness regarding authorship guidelines [19]. Moreover, in Saudi Arabia, most of the researchers and health care providers are non-native English speakers, who tend to have difficulty writing in a clear, concise, and credible manner to meet journal requirements [19].

Assessment of knowledge and attitude is useful for identifying gaps and barriers pertaining to a specific problem and thus conducting effective interventions [20-22]. Several studies have been conducted in different regions to examine the attitudes toward proofreading applications and professional medical writers [23-25]. However, there is limited research related to healthcare professionals' knowledge, attitude, and perception toward PMWS services within Saudi Arabia. Information regarding PMWS can help promote scientific publications with high ethical integrity in Saudi Arabia.

Thus, the aim of this study, our primary objective, was to examine the knowledge, attitudes, and perceptions toward PMWS among healthcare professionals at King Fahad Medical City (KFMC). Specifically, we aimed to assess the level of knowledge regarding PMWS among participants and explore any associations between socio-demographic factors and knowledge levels. Additionally, we sought to investigate participants' attitudes toward PMWS, including their perceptions of its value and ethical considerations. By addressing these objectives, we aimed to provide valuable insights into the utilization and perception of PMWS among healthcare professionals in Saudi Arabia.

## Materials and methods

Methodology

Study Design and Setting

An observational cross-sectional study was conducted between August 2019 and August 2020 at KFMC, Riyadh, Saudi Arabia.

Study Participants and Recruitment

Study participants consisted of healthcare professionals from different medical and biomedical science fields in KFMC. Non-healthcare professionals were excluded from the study. A total of 200 healthcare professionals from different departments such as pharmacy, pathology, radiology, etc. were randomly selected and invited to participate in the study.

Sample Size Calculation

The sample size was calculated by using the following formula: n=[{(2k/(k-1))(Zα/2+Zβ)2}/ln(ẟ)2]+2 (formula by Bonett), where ẟ=(1-CA0)/(1-CA1), CA0=the value of Cronbach’s alpha at null hypothesis=0.702, CA1=the expected value of Cronbach’s alpha=0.80, n=sample size, Zα/2=level of confidence (2-sided 95% confidence interval=1.96), Zβ=1.28 for power 90%, and K=number of the items. By substituting into the above formula, n=139. So the minimum required sample size is 139; sample size (with 10% dropout)=155. Therefore, 200 questionnaires were distributed to overcome any non-response in the survey [26,27].

Study tool

Questionnaire Development

The survey questions were designed by experts in this field. A pilot study was conducted among 30 participants affiliated with KFMC and had recently published an article and were selected from the Web of Science database. The reliability of the questionnaire was confirmed using the internal consistency method with Cronbach's alpha coefficient. The 30 items were deemed fit to be loaded for this study, the null hypothesis for which CA0=0.702 (the adjusted Cronbach alpha derived for the pilot sample), and the alternative hypothesis for which CA1=0.80. The content validity of the questionnaire was assessed and reviewed independently by three expert consultants with a background in medicine and biomedical science and were experienced in scientific writing.

Questionnaire

A questionnaire in the English language was used to collect information and consisted of four sections. The first section consisted of five questions related to socio-demographic information including age, gender, academic qualification, years of experience in research, and current position. The second section was related to the knowledge domain and nine questions were asked related to knowledge of PMWS services. The third and fourth sections consisted of 10 and 11 questions related to the attitude and perception toward PMWS services, respectively. These questions were designed to understand the knowledge, common practice, what is defined to be acceptable as standards, and practices.

The combined measure of the dimensions knowledge, attitude, and perception was rated on a uniform 5-point Likert scale (strongly disagree=1, disagree=2, neutral=3, agree=4, and strongly agree=5) for all questions in the three main groups and presented as mean data with standard deviations [28,29]. An online, self-administered survey was filled out by the participants using the researcher’s iPad.

Statistical Analysis

Statistical analysis was performed using SPSS version 25.0 (IBM Corporation, Armonk, NY, US) and MS Excel 16.0 software. Continuous variables were expressed as mean ± standard deviation and categorical variables were expressed as percentages. The normality of the mean scores was checked by the Shiprov-Wilk and Kolmogorov-Smirnov tests. Mann-Whitney test and Kruskal-Wallis test were used for continuous variables without normal distribution. A p-value <0.05 was considered statistically significant.

Ethical Considerations

Ethical approval (19-225) was obtained from the Institutional Review Board at KFMC, Riyadh, Saudi Arabia before collecting the data. Informed consent was sought and the participants signed the consent form before answering the questionnaire. The enrolment of each participant and the identity of the participant were kept confidential as we did not collect any identification information.

## Results

A total of 162 participants out of 200 healthcare professionals who received the questionnaire responded completely, which was more than the minimum required sample size. Table 1 shows the characteristics of the participants. The majority of the participants were between 25 and 45 years old (76.5%) and there was a slight male preponderance (54.9%). The current positions held by the respondents were faculty or consultant (60, 37%), residents (29, 17.7%), specialists/fellows (40, 24.7%), and technicians (33, 20.4%). Over half the respondents (52.5%) had academic qualifications of a master's or a PhD. A total of 92 respondents (56.8%) had been in research for more than three years.

**Table 1:** Demographic characteristics of the participants (N=162)

Characteristic		n (%)
Age (years)	18-25	14 (8.6)
	25-45	124 (76.5)
	45 and above	24 (14.8)
Gender	Female	73 (45.1)
	Male	89 (54.9)
Academic qualifications held	Diploma/bachelor	57 (35.2)
	Master	41 (25.3)
	PhD	44 (27.2)
	Other	20 (12.3)
Numbers of years in research	None	18 (11.1)
	1-3 Years	52 (32.1)
	3-5 Years	41 (25.3)
	More than 5 years	51 (31.5)
Current position	Faculty/consultant	60 (37.0)
	Residents	29 (17.7)
	Specialist/fellow	40 (24.7)
	Technician	33 (20.4)

Table 2 shows the participants' responses and mean scores for the questions related to knowledge, attitude, and perception. The majority of the participants have a high agreement with the statement "medical and scientific writing requires serious effort" and "medical and scientific writing requires frequent practice." Participants also “strongly agreed” that "more studies need to be done in this field." Respondents "agreed" that they had "written a thesis, dissertation or manuscript before," and "medical and scientific writing requires a special talent." In contrast, they have a high disagreement with the statement "I have never been involved in medical and scientific writing before" and "Professional medical writing does not provide real added value." 

**Table 2:** Participants' responses and the mean score for the questions of knowledge, attitude, and perception Data represented as N, %, and mean ± SD. *Likert scale with 5 points was used (strongly disagree=1, disagree=2, neutral=3, agree=4, and strongly agree=5)

Question	Strongly disagree	Disagree	Neutral	Agree	Strongly agree	Mean^* ^± SD
Knowledge	N (%)	N (%)	N (%)	N (%)	N (%)	
I have never been involved in medical and scientific writing before.	67 (41.4)	48 (29.6)	22 (13.6)	17 (10.5)	8 (4.9)	2.1±1.2
I have written a thesis, dissertation, or manuscript before.	11 (6.8)	11 (6.8)	22 (13.6)	52 (32.1)	66 (40.7)	3.9±1.2
I consider myself experienced in publishing research papers.	16 (9.9)	26 (16.0)	55 (34.0)	42 (25.9)	23 (14.2)	3.2±1.2
Medical and scientific writing requires serious effort.	3 (1.9)	0 (0)	12 (7.4)	59 (36.4)	88 (54.3)	4.4±0.8
Medical and scientific writing requires a special talent.	3 (1.9)	17 (10.5)	33 (20.4)	73 (45.1)	36 (22.2)	3.8±1
Medical and scientific writing requires frequent practice.	2 (1.2)	0 (0)	15 (9.3)	77 (47.5)	68 (42.0)	4.3±0.7
I have previously taken a course in writing.	20 (12.3)	38 (23.5)	21 (13.0)	60 (37.0)	23 (14.2)	3.2±1.3
I have previously used proofreading applications.	27 (16.7)	38 (23.5)	30 (18.5)	46 (28.4)	21 (13.0)	3±1.3
I am familiar with the concept of ghostwriting.	26 (16.0)	41 (25.3)	30 (18.5)	46 (28.4)	19 (11.7)	2.9±1.3
Attitude						
It is acceptable for professional medical writers to provide me with assistance in the preparation of a publication.	6 (3.7)	9 (5.6)	20 (12.3)	94 (58.0)	33 (20.4)	3.9±0.9
It is justified to use professional medical writers to write my research papers in preparation of the publication.	12 (7.4)	27 (16.7)	30 (18.5)	70 (43.2)	23 (14.2)	3.4±1.1
I value the assistance of professional medical writers in supporting me with the preparation of publications.	6 (3.7)	7 (4.3)	24 (14.8)	80 (49.4)	45 (27.8)	3.9±1
Professional medical writers' charges are very expensive.	2 (1.2)	6 (3.7)	72 (44.4)	57 (35.2)	25 (15.4)	3.6±0.8
The use of professional medical writing is considered to be “unethical.”	30 (18.5)	67 (41.4)	45 (27.8)	12 (7.4)	8 (4.9)	2.4±1
Professional medical writers should be recognized for the work they perform in supporting me with the preparation of a publication.	5 (3.1)	10 (6.2)	32 (19.8)	82 (50.6)	33 (20.4)	3.8±0.9
Professional medical writers should not be acknowledged as they do not participate in developing the scientific content or carrying out the research.	27 (16.7)	54 (33.3)	41 (25.3)	27 (16.7)	13 (8.0)	2.7±1.2
Online proofreading applications are equal to medical and scientific writers.	20 (12.3)	48 (29.6)	56 (34.6)	34 (21.0)	4 (2.5)	2.7±1
Professional medical writing is a practice of ghostwriting.	8 (4.9)	39 (24.1)	87 (53.7)	21 (13.0)	7 (4.3)	2.9±0.9
Professional medical writing does not provide real added value.	37 (22.8)	74 (45.7)	31 (19.1)	16 (9.9)	4 (2.5)	2.2±1
Perception						
I face difficulties when I write medical or scientific papers.	12 (7.4)	25 (15.4)	31 (19.1)	77 (47.5)	17 (10.5)	3.4±1.1
Occasionally, it is justified to copy a sentence or two just to get inspiration for further writing.	29 (17.9)	32 (19.8)	52 (32.1)	44 (27.2)	5 (3.1)	2.8±1.1
Writing a scientific paper without plagiarizing is not possible.	32 (19.8)	51 (31.5)	42 (25.9)	26 (16.0)	11 (6.8)	2.6±1.2
I need to ask someone to revise and/or edit my writing.	1 (0.6)	11 (6.8)	34 (21.0)	76 (46.9)	40 (24.7)	3.9±0.9
I have experience of working with professional medical writers in the preparation of manuscripts, abstracts, or proposals.	23 (14.2)	25 (15.4)	32 (19.8)	59 (36.4)	23 (14.2)	3.2±1.3
I use professional medical writers in editing for grammar, spelling, journal-style (including referencing), etc.	18 (11.1)	25 (15.4)	25 (15.4)	73 (45.1)	21 (13.0)	3.3±1.2
I am an expert in medical and scientific writing; however, I ask professional medical writers for the management of time.	18 (11.1)	42 (25.9)	50 (30.9)	42 (25.9)	10 (6.2)	2.9±1.1
It is justified to use professional medical writers in managing reviewers' comments.	11 (6.8)	17 (10.5)	47 (29.0)	71 (43.8)	16 (9.9)	3.4±1
I cannot publish without the use of professional medical writers.	23 (14.2)	41 (25.3)	43 (26.5)	44 (27.2)	11 (6.8)	2.9±1.2
Medical writers have helped effectively to publish my research.	20 (12.3)	29 (17.9)	38 (23.5)	52 (32.1)	23 (14.2)	3.2±1.2
I encourage more studies to be done in this field.	2 (1.2)	4 (2.5)	29 (17.9)	72 (44.4)	55 (34.0)	4.1±0.9

Table 3 shows the association between the mean total score of knowledge and the socio-demographic characteristics of the participants. The mean total score of knowledge was 32.59 out of 45. The age of 45 years and above have a higher significant level of knowledge with a mean score of 35.58±5.55 (p<0.001). The participants with PhD degrees have a higher significant level of knowledge with a mean score of 33.84±5.17 (p=0.044). The participants with more than five years of research experience have a higher significant level of knowledge with a mean score of 35.43±4.64 (p<0.001). The current position of faculty/consultant has a higher significant level of knowledge with a mean score of 34.25±5.00 (p=0.005).

**Table 3:** Association between the mean of total score of knowledge and the socio-demographic characteristics of the participants Data represented as N, %, and mean ± SD; Likert scale with 5 points was used (strongly disagree=1, disagree=2, neutral=3, agree=4, strongly agree=5) for nine questions of the knowledge with a minimum score of 9 and a maximum score of 45. A high score indicates high knowledge. ^*^Significant p-value of <0.05 ^#^Out of 45

		Mean ^#^	SD	P value
Age	18-25	27.14	4.54	<0.001*
	25-45	32.62	5.00	
	45 and above	35.58	5.55	
Gender	Male	32.22	5.54	0.067
	Female	33.32	4.95	
Academics	Diploma/bachelor	31.23	5.65	0.044*
	Master	33.46	5.01	
	PhD	33.84	5.17	
	Other	31.90	5.28	
Research experience	None	27.06	5.68	<0.001*
	1-3 years	30.48	4.01	
	3-5 years	34.15	4.87	
	More than 5 years	35.43	4.64	
Current position	Faculty/consultant	34.25	5.00	0.005*
	Residents	30.17	3.36	
	Specialist/fellow	32.60	5.21	
	Technician	31.67	6.79	
Total score of knowledge		32.59	5.39	

Table 4 shows the association between the mean total score of attitude and the socio-demographic characteristics of the participants. The mean total score of knowledge was 32.23 out of 50. All socio-demographic characteristics of the participants showed no significant differences in relation to the mean total score of attitude.

**Table 4:** Association between the mean of total score of attitude and the socio-demographic characteristics of the participants Data represented as N, %, and mean ± SD; Likert scale with 5 points was used (strongly disagree=1, disagree=2, neutral=3, agree=4, strongly agree=5) for 10 questions of the attitude with a minimum score of 10 and a maximum score of 50. A high score indicates a high attitude. Significant p-value: <0.05. ^#^Out of 50

		Mean^#^	SD	p-value
Age	18-25	30.93	3.00	0.094
	25-45	32.30	3.41	
	45 and above	32.67	2.70	
Gender	Male	32.03	3.52	0.239
	Female	32.54	3.02	
Academics	Diploma/bachelor	32.49	2.72	0.913
	Master	32.05	3.58	
	PhD	32.18	3.77	
	Other	32.00	3.28	
Research experience	None	31.06	2.53	0.098
	1-3 years	32.19	2.96	
	3-5 years	31.80	3.54	
	More than 5 years	33.04	3.53	
Current position	Faculty/consultant	32.57	3.23	0.842
	Residents	32.07	2.76	
	Specialist/fellow	32.08	3.94	
	Technician	31.97	3.07	
Total score of attitude		32.23	3.29	

Table 5 shows the association between the mean total score of perception and the socio-demographic characteristics of the participants. The mean total score of perception was 35.57 out of 55. The participants with more than five years of research experience were found to have a higher significant level of perception with a mean score of 36.84±3.85 (p=0.004). Other socio-demographic characteristics did not show significant differences in relation to the mean total score of perception. 

**Table 5:** Association between the total mean score of perception and the socio-demographic characteristics of the participants Data represented as N, %, and mean ± SD; Likert scale with 5 points was used (strongly disagree=1, disagree=2, neutral=3, agree=4, strongly agree=5) for 11 questions of the knowledge with a minimum score of 11 and a maximum score of 55. A high score indicates a high perception, ^*^Significant p-value of <0.05 ^#^Out of 55

		Mean^#^	SD	p-value
Age	18-25	34.00	2.83	0.076
	25-45	35.40	4.01	
	45 and above	37.33	4.57	
Gender	Male	35.70	4.28	0.510
	Female	35.47	3.83	
Academics	Diploma/bachelor	35.33	3.59	0.574
	Master	35.39	3.26	
	PhD	36.11	4.38	
	Other	35.40	5.99	
Research experience	None	33.83	3.22	0.004*
	1-3 years	34.38	4.13	
	3-5 years	36.24	4.05	
	More than 5 years	36.84	3.85	
Current position	Faculty/consultant	36.25	4.53	0.387
	Residents	35.07	3.52	
	Specialist/fellow	34.75	4.09	
	Technician	35.76	3.54	
Total score of perception		35.57	4.08	

## Discussion

This study aimed to examine the knowledge, attitude, and perception toward PMWS among healthcare professionals at KFMC. In the current study, it was found that socio-demographic factors of the participants including age of ≥45 years, those with a PhD degree, >5 years of research experience, and current position of faculty/consultant showed a significantly higher level of knowledge related to PMWS services. Relevantly, a study carried out among undergraduate students in Jouf suggested that knowledge related to scientific writing misconduct such as plagiarism increases as students move up the academic ladder [30]. Taken together, this suggests the need for academic and research institutes to take measures that target young students and professionals to improve their awareness regarding steps to uphold ethical practices in research and scientific writing.

Moreover, our study found a significantly higher level of perception related to PMWS among participants with more than five years of research experience (p=0.004). This can be reasoned based on the findings of our study where high knowledge levels were significantly associated with senior-level professionals. Since knowledge affects perception, the higher knowledge of the senior and experienced professionals may presumably influence their higher perception regarding PMWS [31]. Moreover, a study was conducted by Das and Das (2018) on Indian surgeons' knowledge and attitudes regarding PMWS where respondents cited a lack of time and support as the key challenges that prevented them from publishing often [24]. Accordingly, the high perception of experienced researchers toward writing services may be presumed concerning the support it offer. The findings from our study are consistent with previously published studies conducted in other areas of the world. We found that the majority (78.4%) of our respondents felt it was acceptable to use PMWS, which echoes the result (82.9%) reported by a study conducted in Oxford [23]. Likewise, 77.2% of our respondents valued the support of professional medical writers compared with (84%) of those surveyed in Oxford [23]. The fact that the Oxford cohort consisted of academics/clinicians who were all in contact with certain medical communication agencies and our survey recipients encompassed people with or without such experience might explain the higher rate of acceptability in that study. Nevertheless, the resemblance in these results suggests that the use of medical writing services is relatively widespread among the health professionals in KFMC, the same as the cohort in Oxford. Gattrell et al. found that medical writing support improves the quality of clinical trial reporting and their adherence to Consolidated Standards of Reporting Trials (CONSORT) [32]. The high level of acceptance of medical writing services of our respondents would allude to the benefits our cohort obtained from PMWS for a written manuscript. However, it is worth pointing out that there is a study that suggested that the use of professional writers did not improve the impact of the published article in terms of annual citations, article views, and altimetric score and did not show any increased adherence to CONSORT-A [33,34]. However, it must be noted that 12.3% of our respondents deemed it unethical to use PMWS. It must be considered that without the aid of medical writers, there would be a large volume of high-quality research from non-native English speakers not being published, leading to a slew of the world's published data in favor of English-speaking countries and throttling of medical advancements made by non-English-speaking researchers. Regarding specific areas of services delivered by PMWS, more help in just editing and grammatical styling (58.1%) was acquired by our respondents than in document preparation (50.4%), which affirms the finding of the Oxford study that the editorial process is the most sought-after help by health care professionals [23].

It is noteworthy that our study revealed alarmingly high ignorance toward ethical guidelines in scientific writing, such that only less than half (40.1%) of our respondents were familiar with the concept of "ghostwriting." Healthcare professionals who do not fully understand the difference between a professional medical writing service and a ghostwriting service might inadvertently become involved with unethical medical writing practices [35,36]. Due to the poor understanding of the concept of ghostwriting among our respondents, we could not accurately look at the prevalence of ghostwriting within KFMC. This raises important ethical issues that need to be addressed, sooner rather than later, by educating healthcare professionals about the difference between professional medical writing services and ghostwriting, as well as the available guidelines for using medical writing services and detecting ghostwriters [37]. Enhanced awareness of how to use medical writing services ethically is vital to the future of medical research [38,39].

To the best of our knowledge, this is the first study to investigate the medical professionals' knowledge and attitudes toward PMWS within a medical institution in Saudi Arabia. Having a thorough survey of the literature made it very clear that there are still gaps in knowledge regarding the healthcare providers' insight into PMWS. Such a study is of paramount importance for the rapidly growing Saudi research community and relevant policymakers. Although we enrolled a wide range of respondents from varying levels of experience and qualifications, with a healthy gender balance, which strengthens the representative power of the data produced, a potential limitation is that the majority of our respondents were aged 25-45 years, which may lead to a biased interpretation of some data. Especially since the study suggested that age is an important parameter that influences how people view PMWS. Another shortcoming that needs to be overcome in future studies is the absence of an objective measure to identify the effectiveness of PMWS in helping healthcare professionals publish their work. Although our respondents described that they valued the input of PMWS, we had no objectively reviewed documents to assess the improvement in writing contributed by such support. Moreover, since most of the respondents in our study have worked with PMWS and given some studies that have indicated that industry-funded manuscripts are more likely to be prepared using professional medical writers, it would be beneficial to know if this holds true in the case of our respondents [40,41].

Based on our findings, it is worth exploring the possibility of designing and implementing special courses and workshops on medical writing as a part of graduate and postgraduate learning. Moreover, the strategic impact of institutional cooperation with medical writing companies should be studied in depth. Normalizing the use of PMWS creates serious ethical considerations within the medical and scientific communities. One of the most important concerns is transparency and honesty. Scientific literature is required to be transparent and correctly represent the contributions of all authors. Ghostwriting weakens this concept by obscuring the genuine authors of a publication, which can lead to data falsification or conflicts of interest. This lack of transparency can undermine faith in the scientific process and jeopardize the integrity of medical research. The rising popularity of professional medical writing assistance in industry-funded papers raises concerns about the independence and autonomy of researchers. Scholars may feel pushed to provide their names to ghostwritten articles to gain future funding or retain professional connections with industry sponsors. This pressure could compromise academic independence and the capacity to perform objective research.

## Conclusions

Our research has shown that PMWS is relatively common in KFMC, with many healthcare professionals considering it valuable. There is generally a positive perception toward these services, with few considering medical writing unethical. However, our survey highlights the lack of a thorough understanding of what precisely PMWS involves. This paper adds nicely to the body of international research and also exposes some uncharted waters in this field for future exploration. Increased awareness of PMWS as a practice, with education for health professionals on the ethical use of medical writing, in particular, the ethical principles underpinning medical writing, would be advantageous. It should be noted that the study was conducted at a single institution (KFMC); therefore, we are not generalizing the conclusions about PMWS use and attitudes to the entire Saudi healthcare. However, increased reliance on PMWS in industry-funded manuscripts heightens concerns about transparency, bias, and academic integrity. Efforts to address these issues should focus on promoting transparency and sustaining academic credibility and reliability of scientific research through a multifaceted approach involving collaboration between researchers, institutions, journals, and industry partners.

## Appendices

Questionnaire knowledge, attitude, and perception toward medical and scientific writing

Dear Participant, Good Day

The following questionnaire is regarding the perception of “Medical and Scientific Writing” among scientists and researchers, specifically, and the scientific community in general. Please answer the 30 questions as accurately as possible. It should be noted that the questionnaire is completely anonymous. In answering each question, please indicate whether you strongly agree, agree, neither agree nor disagree, disagree, or strongly disagree with the statement in the question. Your patience and cooperation are highly appreciated. Thank you!

i. Age

o 18-25 o 25-45 o 45 and above

ii. Gender

o Female o Male

iii. Academic Qualifications Held

o Diploma/bachelor o Master o PhD o Other

iv. Number of Years in Research

o None o 1-3 years o 3-5 years o More than 5 years

v. Current Position

o Technician o Residents o Specialist/fellow o Assistant professor/assistant consultant o Associate professor/consultant o Professor

1. This is the first time I know about medical and scientific writing. o Strongly disagree o Disagree o Neutral o Agree o Strongly agree

2. I have written thesis, dissertation, or manuscript before. o Strongly disagree o Disagree o Neutral o Agree o Strongly agree

3. I consider myself experienced in publishing research papers. o Strongly disagree o Disagree o Neutral o Agree o Strongly agree

4 Medical and scientific writing requires serious effort. o Strongly disagree o Disagree o Neutral o Agree o Strongly agree

5. Medical and scientific writing requires a special talent. o Strongly disagree o Disagree o Neutral o Agree o Strongly agree

6. Medical and scientific writing requires frequent practice. o Strongly disagree o Disagree o Neutral o Agree o Strongly agree

7. I have previously taken a course in writing. o Strongly disagree o Disagree o Neutral o Agree o Strongly agree

8. I have previously used proofreading applications. o Strongly disagree o Disagree o Neutral o Agree o Strongly agree

9. I am familiar with the concept of ghostwriting. o Strongly disagree o Disagree o Neutral o Agree o Strongly agree

10. It is acceptable for professional medical writers to provide me with assistance in the preparation of a publication. o Strongly disagree o Disagree o Neutral o Agree o Strongly agree

11. It is justified to use professional medical writers to write my research papers in preparation of a publication. o Strongly disagree o Disagree o Neutral o Agree o Strongly agree

12. I value the assistance of professional medical writers in supporting me with the preparation of publications. o Strongly disagree o Disagree o Neutral o Agree o Strongly agree

13. Professional medical writers’ charges are very expensive. o Strongly disagree o Disagree o Neutral o Agree o Strongly agree

14. The use of professional medical writing is considered unethical. o Strongly disagree o Disagree o Neutral o Agree o Strongly agree

15. Professional medical writers should be recognized for the work they perform in supporting me with the preparation of a publication. o Strongly disagree o Disagree o Neutral o Agree o Strongly agree

16. Professional medical writers should not be acknowledged as they do not participate in developing the scientific content or carrying out the research. o Strongly disagree o Disagree o Neutral o Agree o Strongly agree

17. Online proofreading applications are equal to medical and scientific writers. o Strongly disagree o Disagree o Neutral o Agree o Strongly agree

18. Professional medical writing is a practice of ghostwriting. o Strongly disagree o Disagree o Neutral o Agree o Strongly agree

19. Professional medical writing does not provide real added value. o Strongly disagree o Disagree o Neutral o Agree o Strongly agree

20. I face difficulties when I write medical or scientific papers. o Strongly disagree o Disagree o Neutral o Agree o Strongly agree

21. Occasionally, It is justified to copy a sentence or two just to get inspiration for further writing. o Strongly disagree o Disagree o Neutral o Agree o Strongly agree

22. Writing a scientific paper without plagiarizing is not possible. o Strongly disagree o Disagree o Neutral o Agree o Strongly agree

23. I need to ask someone to revise and/or edit my writing. o Strongly disagree o Disagree o Neutral o Agree o Strongly agree

24. I have experience of working with professional medical writers in the preparation of manuscripts, abstracts, or proposals. o Strongly disagree o Disagree o Neutral o Agree o Strongly agree

25. I use professional medical writers in editing for grammar, spelling, journal style (including referencing), etc. o Strongly disagree o Disagree o Neutral o Agree o Strongly agree

26. I am an expert in medical and scientific writing; however, I ask professional medical writers for management of time. o Strongly disagree o Disagree o Neutral o Agree o Strongly agree

27. It is justified to use professional medical writers in managing reviewers’ comments. o Strongly disagree o Disagree o Neutral o Agree o Strongly agree

28. I cannot publish without the use of professional medical writers. o Strongly disagree o Disagree o Neutral o Agree o Strongly agree

29. Medical writers have helped effectively to publish my research. o Strongly disagree o Disagree o Neutral o Agree o Strongly agree

30. I encourage more studies to be done in this field. o Strongly disagree o Disagree o Neutral o Agree o Strongly agree
